# Antidiabetic Activity of Polysaccharides from Tuberous Root of *Liriope spicata* var. *prolifera* in KKAy Mice

**DOI:** 10.1155/2013/349790

**Published:** 2013-05-26

**Authors:** Yihui Liu, Luosheng Wan, Zuoqi Xiao, Jingjing Wang, Yonglong Wang, Jiachun Chen

**Affiliations:** Hubei Key Laboratory of Natural Medicinal Chemistry and Resource Evaluation, Tongji School of Pharmaceutical Sciences, Huazhong University of Science and Technology, Hangkong Road 13, Wuhan 430030, China

## Abstract

Tuberous root of *Liriope spicata* var. *prolifera* has been widely used as a traditional Chinese medicine for the treatment of diabetes. The present study investigated the antidiabetic effect and the potential mechanisms of two new polysaccharides (LSP1, LSP2) and the total polysaccharides (TLSP), isolated from the tuberous roots. Upon the intragastric administration in obese insulin-resistant diabetic KKAy mice for 28 days, TLSP, LSP1, and LSP2 all caused a remarkable decrease of fasting blood glucose and significant improvement of insulin resistance and serum lipid metabolism in diabetic mice. In addition, liver histological analysis showed that TLSP, LSP1, and LSP2 significantly ameliorated the hepatocyte hypertrophy and decreased the lipid accumulation in the mice liver. Further experiments suggested that TLSP, LSP1, and LSP2 effectively inhibited hepatic gluconeogenesis and increased hepatic glycolysis and hepatic glycogen content. Furthermore, the mechanistic analysis showed the increased expression of insulin-receptor **α** subunit, insulin-receptor substrate-1, phosphatidylinositol 3-kinase, and peroxisome proliferators-activated receptors **γ**. These results suggested that TLSP, LSP1, and LSP2 manifest strong antidiabetic activity, therefore hold a great promise for therapeutic application in diabetic therapy and other related metabolic disorders.

## 1. Introduction

Diabetes has been a life-threatening metabolic disorder affecting countless patients worldwide, particularly in China [1]. About 90% of diabetic populations are diagnosed with type 2 diabetes [2]. Due to the limited and undesirable side effects of the commonly used antidiabetic drugs, such as metformin, thiazolidinediones, sulphonylureas, and *α*-glucosidase inhibitors, many efforts have been made in search for complementary and alternative therapeutic agents from herbal medicine [3]. 

Insulin resistance is a major obstacle in the diabetes treatment and is often accompanied with hyperglycemia, hyperinsulinemia, and hyperlipaemia in obesity-induced type 2 diabetic patients, which is also regarded as one of the risk factors leading to a series of complications, such as nephropathy, retinopathy, myocardial infarction, and neuropathy [4]. Defects in molecules of the insulin signaling pathway are now thought to be a major mechanism involved in the development of insulin resistance. Insulin has been proved to initiate its signaling through insulin receptor (InsR-*α* and -*β*) and then stimulate tyrosine phosphorylation of insulin receptor substrate (IRS) proteins, which activate phosphatidylinositol 3-kinase (PI3K) in turn [5–9]. Furthermore, the activation of PI3K has been shown to be essential for the metabolic effect of insulin and insulin-induced glycogen synthesis [10–14]. As a member of the nuclear hormone receptor superfamily, peroxisome proliferators-activated receptors *γ* (PPAR*γ*) is expressed in adipocytes, liver, muscle, and other tissues and is involved in glucose homeostasis, lipid storage, and adipocytokine regulation [15, 16]. Thus, the activity of InsR, IRS, PI3K, and PPAR*γ* is important in mediating the metabolic effects of insulin and closely correlative with insulin resistance.

Here, we reported the antidiabetic activity from the tuberous root of *Liriope spicata* var. *prolifera* which is indigenous to Hubei province, China. It was recorded as “Suizhou Maimendong” in “Zhenglei Bencao,” a medical classic in the Song Dynasty and as Liriopes Radix in Pharmacopoeia of China, frequently used as “maidong” in prescriptions for the treatment of diabetes. We demonstrate that the water extract and crude polysaccharides derived from the tuberous root of *Liriope spicata* var. *prolifera* improve hyperglycemia and insulin resistance in low-dose–streptozotocin (STZ)-induced diabetic BABL/c mice. Two-novel water-soluble polysaccharides, named LSP1 and LSP2, were considered to be the active components with significant anti-diabetic properties [17–19]. 

Our study further evaluates the anti-diabetic activity of the LSP1, LSP2, and the total polysaccharides (TLSP) from the tuberous root of *Liriope spicata* var. *prolifera* in vivo using genetic obese diabetic KKAy mice. These KKAy mice are developed by transferring the yellow Ay gene into the KK strain, which present severe hyperglycemia, hyperinsulinemia, glucose intolerance, and obesity by 56 days of age. And they are widely used for evaluating antidiabetic and antiobesity agents [20]. So, in the present study, we used this mice model to evaluate the effect of the polysaccharides on hyperglycemia, hyperlipidemia, and insulin resistance. Moreover, we reveal the molecular mechanism whereby anti-diabetic activity of the polysaccharides is mediated by the activation of InsR-*α*/IRS-1/PI3K signaling pathway, the expression of PPAR*γ*, and the regulation of glucose metabolism, resulting in the decrease of blood glucose concentrations of these treated mice.

## 2. Materials and Methods

### 2.1. Preparation of Polysaccharides

**Figure 1:**
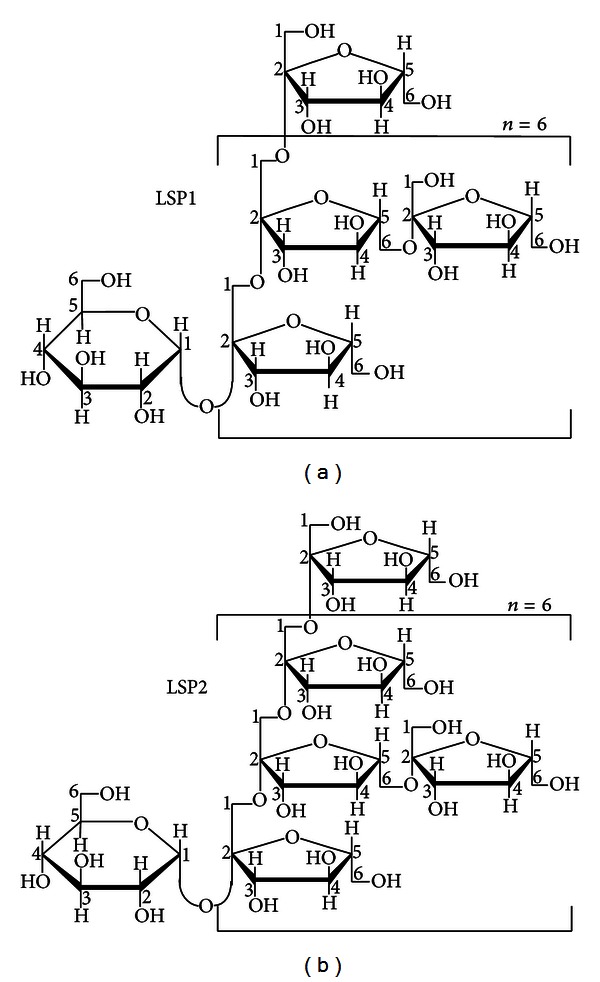
Chemical structure of LSP1 (a) and LSP2 (b).

The dried tuberous root of *Liriope spicata* var. *prolifera* was obtained from Xiangfan city, Hubei province, China, in April 2006. The crude polysaccharides were obtained from the tuberous root according to the previous report [12]. Then, the crude polysaccharides were applied to a diethylaminoethyl cellulose 52 (DEAE-cellulose 52) column (2.5 × 38 cm). The fraction eluted with H_2_O was collected and lyophilized to obtain the total polysaccharides (TLSP, the yield was 19.3 g/100 g of crude material). TLSP was further purified on an AB-8 macroporous resin column (2.5 × 70 cm). Two white purified polysaccharide fractions were collected and lyophilized, named LSP1 (the yield was 4.8 g/100 g of crude material) and LSP2 (12.3 g/100 g), respectively. The dried TLSP, LSP1 and LSP2 were dissolved in distilled water, at concentrations of 10, 20 mg/ml for oral administration, respectively. LSP1, and LSP2 were both small molecule polysaccharides with molecular weight of 3.20 and 4.29 kD, respectively. The chemical structures (Figure 1) of LSP1 and LSP2 were determined by various chemical and spectral methods [14].

### 2.2. Animals and Experimental Design

Male KKAy mice and C57BL/6J mice (6 weeks old) were purchased from Laboratory Animal Center of Chinese Academy of Medical Sciences, Beijing, China. Animals were housed at a temperature of 25 ± 2°C and 55% ± 5% relative humidity on a light/dark cycle of 12 ± 1 h with *ad libitum* access to food and water. The KKAy mice were fed with high-fat diets (HFD; consisting of 10% fat, 20% fructose, 10% egg, and 60% basic diet (w/w)) for 28 days to induce insulin resistance. Diabetes was induced successfully when the fasting blood glucose (FBG) level of KKAy mouse was higher than 11.1 mmol/L [21, 22]. The KKAy mice were served as type 2 diabetes mice, while C57BL/6J mice with normal diets were used as normal control [23–25]. The study had been carried out in compliance with the Principles of Laboratory Animal Care published by the National Institutes of Health, as approved by the Ethical Committee of Huazhong University of Science and Technology. All of the animals were treated with humane care throughout the experiment and performed surgery under anesthesia.

The KKAy diabetic mice received daily intragastric administration of polysaccharides (TLSP, LSP1, and LSP2) at doses of 100 and 200 mg·kg^−1^·day^−1^ and rosiglitazone at a 2 mg·kg^−1^·day^−1^ (all the drugs were dissolved in physiological NaCl-solution which was used as vehicle) for 28 days, while C57BL/6J normal control and KKAy diabetic control were only administrated with vehicle, respectively. All KKAy diabetic mice were continuously fed with high-fat diets, and the C57BL/6J mice were fed with normal diets till the end of the study [21]. 

### 2.3. Measurement of Body Weight, Food Intake, FBG, FINS, Lipid Levels, and OGTT

FBG levels were estimated at 7 days interval by using one touch glucometer (JPS-7, Yicheng, Beijing). FINS levels were determined by using an insulin radioimmunoassay kit (Beijing North Institute of Biological Technology, Beijing) at day 28. The index of homeostasis model assessment of insulin resistance (HOMA-IR) was calculated as follows: FBG [mmol/L] × FINS [mIU/L]/22.5. Blood samples for evaluation of total cholesterol (TC), high density lipoprotein cholesterol (HDL), low density lipoprotein cholesterol (LDL), and triglyceride (TG) were taken from the vein behind the eye sockets and were spectrophotometrically determined at day 28 as previously described [26, 27]. The OGTT was performed on day 14: mice were given D-glucose (50 mmol/kg) after 60 min of drug administration, and the blood glucose levels were measured from tail blood samples immediately at 0, 30, 60, and 120 min after the glucose loaded.

### 2.4. Examination of Histopathology and Immunohistochemistry

Histological samples of liver were resected and fixed in 4% paraformaldehyde (10% formaldehyde phosphate buffered saline, pH 7.4) for 12 hours, then embedded in paraffin using a tissue processor. After paraformaldehyde fixation, the liver tissues sections were taken in 4 *μ*m thickness, stained with hematoxylin-eosin reagent. The sectional areas of liver tissue were analyzed for the purpose of quantifying the lipid accumulation in the liver. Five to seven sections were taken for each KKAy mice, and five fields were randomly selected in each liver sections to calculate the rate of lipid accumulation area in each section by an image analysis system Image Pro Plus (Version 5.0 Media Cybernetics, Silver Spring, MD) [28]. To perform immunohistochemistry, 4-*μ*m-thick liver tissue sections were deparaffinized and quenched with 0.6% hydrogen peroxide for 15 minutes. Then the slides were incubated with primary antibody for 1 h at 20 ± 2°C. The sections were rinsed in Tris-buffered saline, incubated with anti-mouse or anti-rabbit secondary antibodies for 30 min, followed by an avidin-biotin-peroxidase conjugate (ABC Elite; Vector) for 30 min. Labeling was detected using a diaminobenzidine (DAB) chromogen solution for 5 min [29, 30]. The primary and secondary antibodies were murine IgG polyclonal antibody (mAb) against PI3K, IRS-1, InsR-*α*, and PPAR*γ* (cell signaling technology). Negative controls were obtained by occulting the primary antibody or by using an unrelated mouse monoclonal antibody. Digital images were captured using a Nikon and determination by either microscopy or morphometry.

### 2.5. Western Blot of InsR-*α*, IRS-1, PI3K, and PPAR*γ*

The concentrations of total protein extracts from liver tissue were quantified by the BCA assay protein kit (Pierce, Rockford, IL). Proteins (30 *μ*g/sample) were lysed in hot Laemmli buffer (95°C) and separated by 10% sodium dodecyl sulfate-polyacrylamide gel electrophoresis (SDS-PAGE) and transferred onto nitrocellulose membrane (Bio-Rad Laboratories, Hercules, CA) [31, 32]. Membranes were blocked for 2 h with 5% skim, milk in Tris-buffered saline containing 0.1% Tween 20 (TBST), and then incubated, respectively with primary antibodies against PI3K, IRS-1, InsR-*α*, and PPAR*γ* (Cell Signaling Technology) for 16 h at 4°C. Membranes were washed for three times in TBST and then incubated with horseradish peroxidase-conjugated anti-rabbit IgG or anti-mouse IgG secondary antibody for 2 h (Amersham Biosciences). Immunodetection was performed according to the ECL Western blotting protocol of Amersham Buchler (Braunschweig, Germany) and quantified by Image Pro Plus (Version 5.0 Media Cybernetics, Silver Spring, MD). Experiments were repeated at least twice.

### 2.6. Measurement of Hepatic Glycogen Content, Glucokinase, and Glucose-6-Phosphatase Activities

The glycogen in liver tissues was extracted by KOH solution, precipitated with ethanol and redissolved in distilled water. Thereafter, the hepatic glycogen content (expressed as mg/g wet tissue) was determined by the anthrone-reagent method and assayed by a spectrophotometer at 620 nm [33]. Glucokinase (GK) activity was measured by using a spectrophotometric method [34, 35]. Briefly, liver tissues were homogenized and centrifuged; the supernatant was used to generate NADPH which was measured by detecting the absorbance at 340 nm. Based on the yield of NADPH, GK activity was estimated by the standard method. Protein concentration was quantified with Bradford reagent, and the activity (mU) was defined as mmol of NADPH generated/min/mg of protein [36, 37]. Glucose-6-phosphatase (G6Pase) activity was assayed by a spectrophotometric method [38]. In brief, glucose-6-phosphate was added to the liver extract and then converted into glucose and inorganic phosphate, by a determined amount of phosphor-molybdous complex at 700 nm. The protein content was also quantified with Bradford reagent. The activity (mU) was expressed as mmol of phosphate released/min/mg of protein [18, 38].

### 2.7. Statistical Analysis

Data were performed using Statistical Package for Social Sciences version 16.0 (SPSS, Chicago, IL). Results are expressed as mean ± SD Student's *t*-test and ANOVA were used for group comparisons, with *P* value < 0.05 considered statistically significant.

## 3. Results 

### 3.1. Effects of TLSP, LSP1, and LSP2 on Body Weight, Food Intake, and FBG

**Figure 2:**
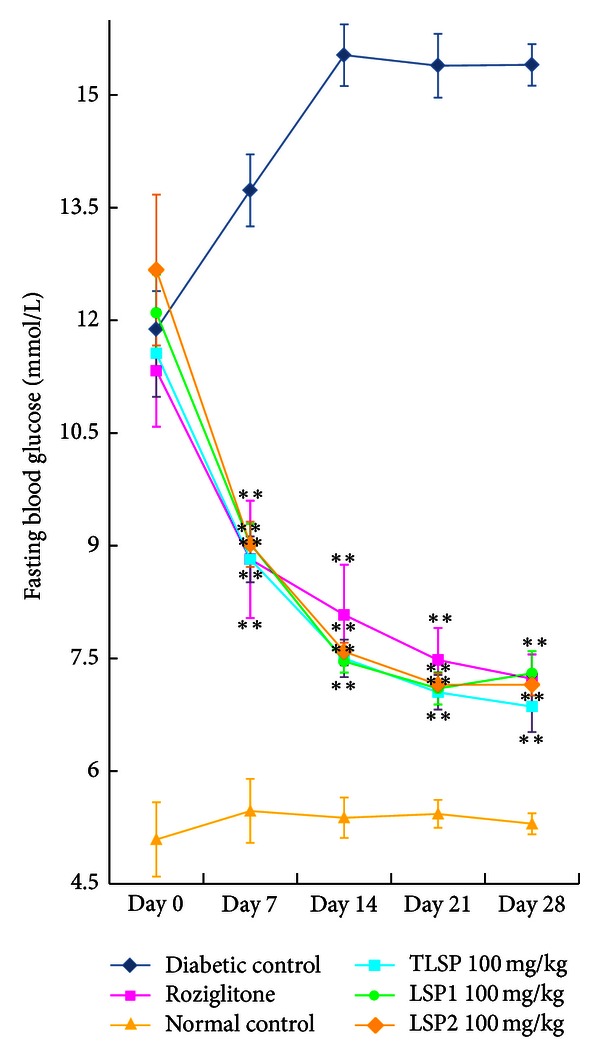
Fasting blood glucose concentrations were measured in obese KKAy mice of insulin resistance. FBG in obese KKAy mice treated with TLSP (100 mg·kg^−1^·day^−1^), LSP1 (100 mg·kg^−1^·day^−1^), LSP2 (100 mg·kg^−1^·day^−1^), normal control C57BL/6J mice, and diabetic control obese KKAy mice and KKAy mice treated with rosiglitazone. Data were presented as mean ± SD (*n* = 7-8). *P < 0.05, **P < 0.01 versus diabetic control (vehicle treated).

Body weight and food intake were significantly increased in KKAy control mice compared with the normal control (vehicle-treated) mice. TLSP, LSP1, LSP2, and rosiglitazone treatment resulted in slight but not significant loss of body weight (about 11% ± 8%,  *P* > 0.05) and food intake (about 16% ± 15%,  *P* > 0.05) during the study. FBG was measured at day 0, 7, 14, and 28. On day 0, all KKAy diabetic control mice had significantly higher FBG levels than normal mice, as shown in Figure 2 and Table 1. After 7 days of administration, FBG decreased significantly in all KKAy diabetic mice treated with 100 and 200 mg·kg^−1^·day^−1^ of TLSP, LSP1, and LSP2 (*P* < 0.01) versus diabetic control, and the FBG levels were stable within the following 21 days. There was no significant difference in FBG levels between all polysaccharides-treated mice and rosiglitazone-treated mice after 28 days administration.

### 3.2. Effects of TLSP, LSP1, and LSP2 on Glucose Tolerance

**Figure 3:**
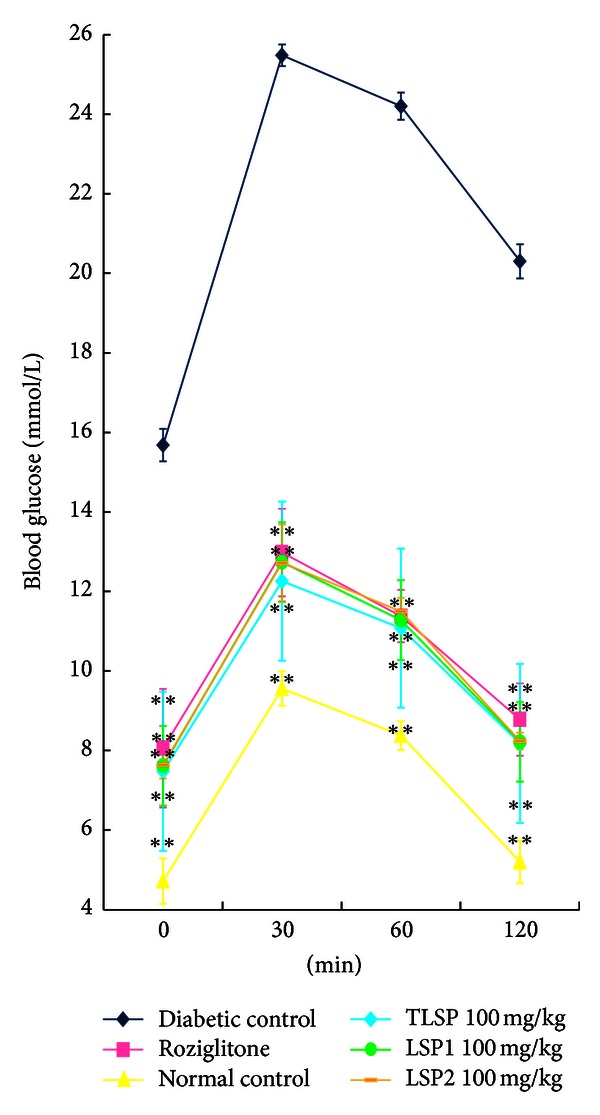
Time course changes in the level of serum glucose during OGTT. OGTT in obese KKAy mice treated with TLSP (100 mg·kg^−1^·day^−1^), LSP1 (100 mg·kg^−1^·day^−1^), LSP2 (100 mg·kg^−1^·day^−1^), normal control C57BL/6J mice and diabetic control obese KKAy mice, and KKAy mice treated with rosiglitazone. Data were presented as mean ± SD (*n* = 7-8). *P < 0.05, **P < 0.01 versus diabetic control (vehicle treated).

To assess glucose homeostasis and insulin sensitivity in mice treated with polysaccharides, OGTT was performed. In Figure 3 and Table 2, the effects of TLSP, LSP1, and LSP2 on oral glucose tolerance in KKAy are presented. The glucose tolerance of diabetic control was severely impaired, compared to normal control (*P* < 0.01). After orally administered with glucose, the rates of increase in the blood glucose level were at the same rate for the drug-treated groups and normal control mice during the first 60 min. After that, the blood glucose level in diabetic control was at a sustained high level while all the drug treated groups showed significant (*P* < 0.01) declined trend in the blood glucose level during the 2 hours study.

### 3.3. Effects of TLSP, LSP1, and LSP2 on FINS and HOMA-IR

The FINS and HOMA-IR of diabetic control (obese diabetic KKAy mice) were much higher, compared with normal control (*P* < 0.01, Table 3). After 28 days of treatment, the FINS levels of mice treated with TLSP, LSP1, LSP2, and rosiglitazone were significantly lowered, compared to diabetic control (*P* < 0.01). And also the HOMA-IR of diabetic mice treated with TLSP, LSP1, and LSP2 was significantly reduced, compared to the diabetic control (*P* < 0.01). 

### 3.4. Effects of TLSP, LSP1, and LSP2 on Lipid Levels in Serum

As shown in Table 4, the TC, TG, HDL, and LDL conditions of obese diabetic KKAy mice were severely impaired, compared with normal control (*P* < 0.01). After 28 days of treatment, the serum TC, TG, and LDL concentrations were significantly lower in obese KKAy mice treated with TLSP, LSP1, and LSP2, compared with diabetic control (*P* < 0.01). However, though the levels of HDL concentrations of TLSP, LSP1, and LSP2 treated mice was not decreased, the ratio of HDL/TC was significantly higher than that of diabetic control (*P* < 0.01). Additionally, the hypolipidemic effects of TLSP, LSP1 and LSP2 on TC, TG, HDL, and LDL levels were more remarkable, compared to rosiglitazone in obese diabetic KKAy mice (*P* < 0.01) [39, 40].

### 3.5. Effects of TLSP, LSP1, and LSP2 on Histopathology of Liver

**Figure 4:**
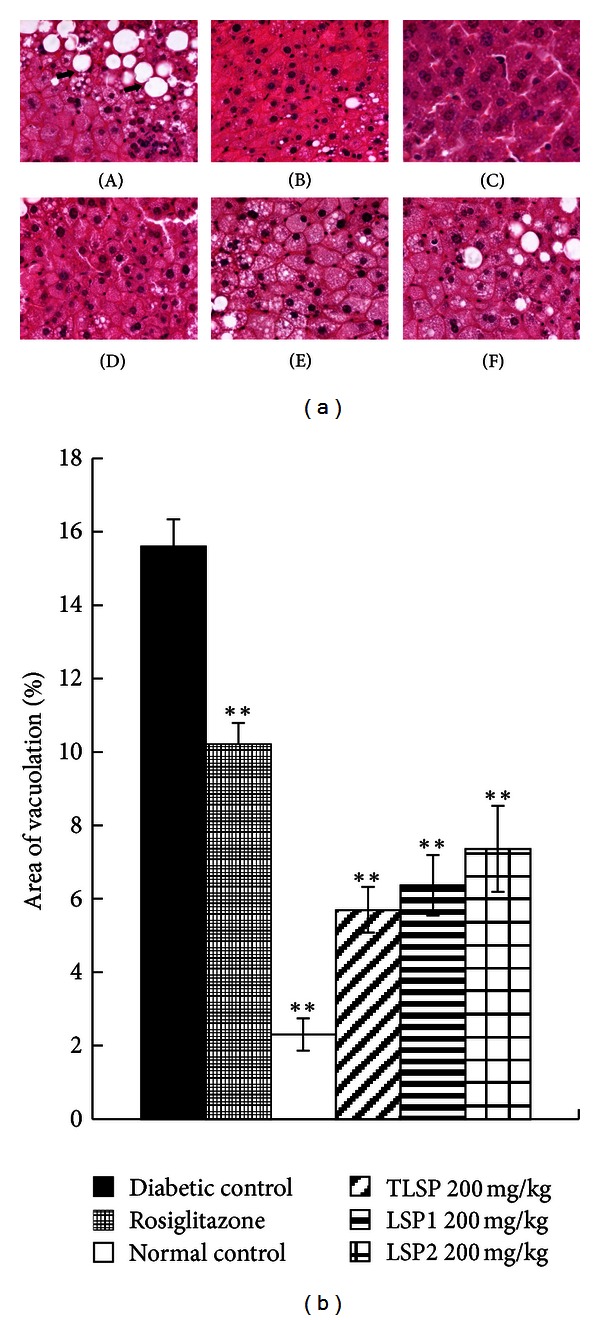
Representative changes of morphology in obese KKAy mice. (a) Photomicrographs of H-E staining of the liver tissue in diabetic KKAy mice. (A) Diabetic control obese KKAy mice; (B) obese KKAy mice treated with rosiglitazone; (C) normal control C57BL/6J mice; (D) obese KKAy mice treated with TLSP (200 mg·kg^−1^·day^−1^); (E) obese KKAy mice treated with LSP1 (200 mg·kg^−1^·day^−1^); (F) obese KKAy mice treated with LSP2 (200 mg·kg^−1^·day^−1^). Severe hepatic steatosis (arrows) was evident in diabetic control obese KKAy mice, which were markedly ameliorated in TLSP, LSP1, and LSP2-treated obese KKAy mice. (b) Quantitative analysis of area vacuoation (%) in obese KKAy mice. Samples were stained with hematoxylin and eosin. Data were presented as mean ± SD (*n* = 7-8). *P < 0.05, **P < 0.01 versus diabetic control (vehicle treated).

The diabetic control KKAy mice showed hypertrophy of hepatocytes and hepatic steatosis (Figure 4(a)), characterized by the accumulation of lipid in hepatic intracellular vesicles and ballooning degeneration of hepatocytes. These changes were relatively mild in obese KKAy mice treated with TLSP, LSP1, and LSP2 (Figure 4(b)). 

### 3.6. Improvement of TLSP, LSP1, and LSP2 on InsR-*α*/IRS-1/PI3K Insulin Signaling Pathway

**Figure 5:**
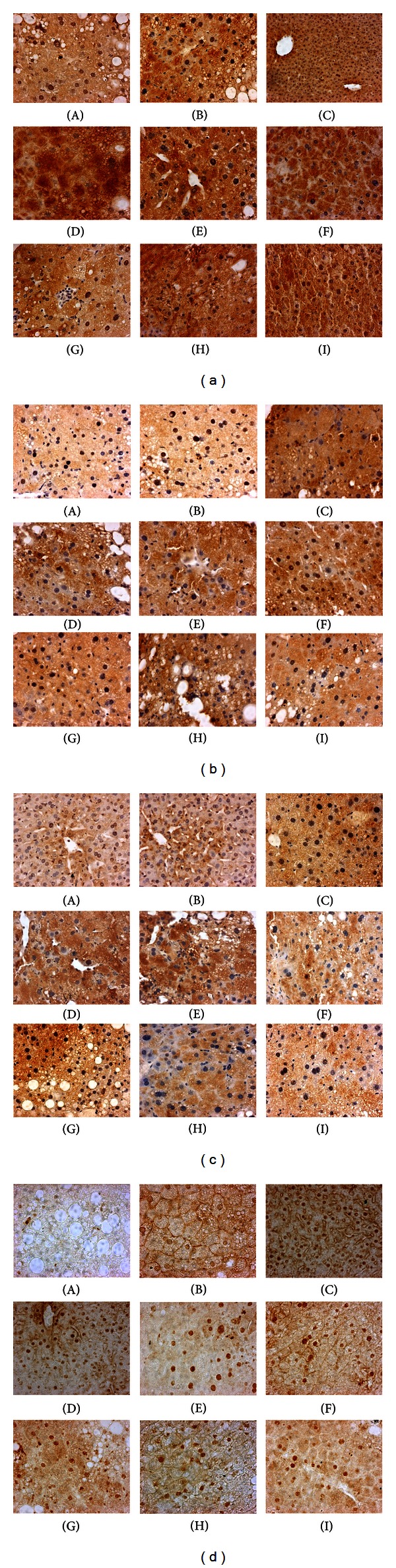
Immunoperoxidase stainings analysis of InsR-*α* (a), IRS-1 (b), PI3K (c), and PPAR*γ* (d) in liver tissues of KKAy mice. Protein expression was mainly expressed in the hepatic cells of the normal control group, which was suppressed in the diabetic control obese KKAy mice. Compared with the diabetic mice, protein levels of polysaccharide treated obese KKAy mice were significantly increased. (A) Diabetic control obese KKAy mice; (B) obese KKAy mice treated with rosiglitazone; (C) normal control C57BL/6J mice; (D)-(E) obese KKAy mice treated with TLSP (200; 100 mg·kg^−1^·day^−1^); (F)-(G) obese KKAy mice treated with LSP1 (200; 100 mg·kg^−1^·day^−1^); (H)-(I) obese KKAy mice treated with LSP2 (200; 100 mg·kg^−1^·day^−1^).

**Figure 6:**
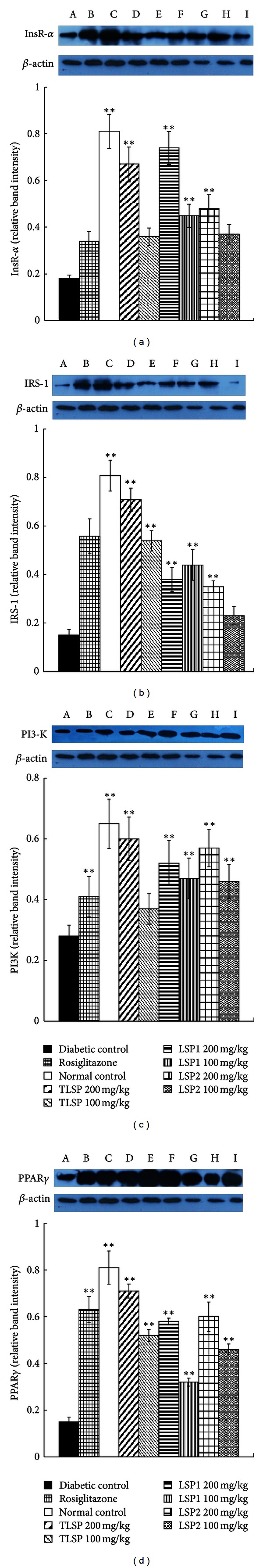
Effect of polysaccharides on protein expression of InsR-*α* (a), IRS-1 (b), PI3K (c) and PPAR*γ* (d) in liver tissues of KKAy mice. (a)–(d) Western blot analysis of InsR-*α*, IRS-1, PI3K, and PPAR*γ*. Expression of InsR-*α*, IRS-1, PI3K, and PPAR*γ* was assessed by western blot analysis. Western blot corrected by that of the protected *β*-actin band in each lane. Each protein is expressed as the intensity of band. Bands were quantified by densitometry. (A) Diabetic control obese KKAy mice; (B) obese KKAy mice treated with rosiglitazone; (C) normal control C57BL/6J mice; (D)-(E) Obese KKAy mice treated with TLSP (200; 100 mg·kg^−1^·day^−1^); (F)-(G) obese KKAy mice treated with LSP1 (200; 100 mg·kg^−1^·day^−1^); (H)-(I) obese KKAy mice treated with LSP2 (200; 100  mg·kg^−1^·day^−1^). Data were presented as mean ± SD (*n* = 7-8). *P < 0.05, **P < 0.01 versus diabetic control (vehicle treated).

The protein expression levels of InsR-*α*, IRS-1, and PI3K in liver tissues of obese diabetic KKAy mice were investigated by immunohistochemistry and western blot. Immunohistochemistry revealed that, compared with normal control, the staining of InsR-*α*, IRS-1, and PI3K in the liver of diabetic control was remarkably diminished. Nonetheless, relative remarkable expression of InsR-*α*, IRS-1, and PI3K were present in the liver of polysaccharides (TLSP, LSP1, and LSP2)-treated mice, compared with diabetic control (Figures 5(a)–5(c)). The western blot analysis revealed that the administration of polysaccharides (TLSP, LSP1, and LSP2, respectively) resulted in a significant elevation in protein expression of PI3K, InsR-*α*, and IRS-1 (Figures 6(a)–6(c)). Thus, both immunohistochemistry and western blot results showed that TLSP, LSP1, and LSP2 could improve insulin signaling transduction of diabetic mice, which resulted in improvement of insulin sensitivity.

### 3.7. Enhancement of TLSP, LSP1, and LSP2 on Protein Level of PPAR*γ*

The protein expression levels of PPAR*γ* in liver tissues of KKAy diabetic mice were also performed by immunohistochemistry and western blot. As shown in Figure 5(d) and Figure 6(d), diabetic mice treated with polysaccharides enhanced the protein levels of PPAR*γ* remarkably, compared with that of diabetic control.

### 3.8. Improvement of TLSP, LSP1, and LSP2 on Hepatic Glucose Metabolism

As shown in Table 5, compared with normal control, G6Pase activity was increased by 86.8% (*P* < 0.01) in diabetic control, yet the GK and hepatic glycogen content activity were decreased by about 70.1% (*P* < 0.01) and 12.4% (*P* < 0.01), respectively. After 28 days of administration, the hepatic G6Pase activities in TLSP, LSP1, and LSP2-treated diabetic mice was significantly reduced, compared to diabetic control (*P* < 0.01). Further, these polysaccharides remarkably elevated the GK activity (*P* < 0.01) and significantly increased the glycogen content (*P* < 0.01). 

## 4. Discussion 

Diabetes is usually called “symptom of wasting-thirst” in traditional Chinese medicine. “Maidong” was recorded in many Chinese ancient medical books for the treatment of wasting-thirst. Till now, it has been used as hypoglycemic agent for centuries in China. 

Our previous study described the antidiabetic properties of two new water-soluble polysaccharides, LSP1 and LSP2, which were isolated from the active crude polysaccharides of the *Liriope spicata* var. *prolifera* tuberous root, in STZ-induced diabetic BALB/c mice [17–19]. The present study was designed to investigate antidiabetic effects and possible mechanisms of TLSP, LSP1, and LSP2 by using KKAy mouse model which is an excellent genetically diabetic model characterized by insulin resistance, hyperglycemia, hyperinsulinemia, and hyperlipaemia that closely resemble obesity-linked type 2 diabetes in humans [41, 42]. Consistent with our previous studies, our present study demonstrated that TLSP, LSP1, and LSP2 caused a remarkable decrease of FBG and FINS levels, as well as a significant improvement of glucose tolerance and serum lipid metabolism in insulin resistance obese diabetic KKAy mice. Further, the results displayed that TLSP, LSP1 and LSP2 significantly enhanced the expression levels of InsR-*α*, IRS-1, and PI3K in liver tissue, which might induce a series of metabolic effects, such as increasing expression of protein involved in glucose transportation and in lipid synthesis. Insulin action contributes to the expression of PI3K, and also insulin is served as a regulator in the expression of lipid oxidation and liquid synthesis genes, suggesting that insulin provides a link between the PI3K and lipid metabolism [43–45]. And further, it is now well known that PPAR*γ* is an important transcriptional regulator of adipogenesis to improve insulin-stimulated glucose disposal by enhancing insulin signaling [46–50]. So, present results suggested that PI3K pathway and PPAR*γ* might be major intermediates in exerting the TLSP, LSP1, and LSP2 mediated effects on glucose and lipid metabolism.

G6Pase, which is abnormally active in insufficient insulin or insulin resistance, catalyzes the last enzymatic reaction that is common to gluconeogenesis and makes the liver releases glucose into the blood, resulting in the rising of blood glucose level [51, 52]. And GK insufficiency, which is caused by insulin insufficient or insulin resistance in diabetes, can cause decreased utilization of glucose for energy production [53]. In the present study, TLSP, LSP1, and LSP2 significantly elevated the GK activity and decreased G6Pase activity to reduce the procession of gluconeogenesis and increase the contents of hepatic glycogen in the liver, which might be due to amelioration of insulin resistance by the polysaccharides.

TLSP, LSP1, and LSP2 remarkably ameliorated hepatic lipid accumulation and ballooning degeneration and inhibited the progression of hepatic steatosis. It might be resulting in the direct protective action on hepatic cells. Thus, TLSP, LSP1, and LSP2 might improve some of the complications of diabetes caused by insulin resistance.

It is interesting to compare the pharmacological effects of TLSP, LSP1, and LSP2 with another insulin-sensitizing agent rosiglitazone. As commonly used agents for type 2 diabetes, thiazolidinedione derivatives (TZDs; including rosiglitazone) have been found to reduce insulin resistance and promote glucose uptake in clinical trials. In addition, PPAR*γ* is considered to mediate the insulin-sensitizing effects of TZDs by regulating a great number of gene transcriptions [54–56]. And in our present study, TLSP, LSP1, LSP2 and rosiglitazone shared common features, such as reduction of FBG and FINS, improvement on glucose tolerance [54, 55], and enhancement on protein expression of PPAR*γ*. However, previous studies have equally shown a positive correlation between the risk of developing ischemic heart disease and raised plasma TC and LDL concentrations [56], while our preparations not only exert their antihyperglycemic effect but also decrease plasma TC, TG, and LDL concentrations and increase the relative HDL levels (HDL/TC) without risk of cardiovascular disease. 

## 5. Conclusion 

The current evidence suggests that the polysaccharides of TLSP, LSP1, and LSP2 isolated from the tuberous root of *Liriope spicata* var. *prolifera*, a traditional Chinese medicine, have shown high hypoglycemic and hypolipidemic activities on insulin-resistant obese diabetic KKAy mice, and the potential mechanisms of action might be due to the alleviation of insulin resistance by improving PI3K signaling pathway, upregulating of protein expression of PPAR*γ*, and improving glucose metabolism. The findings provide convincing evidences that small molecule polysaccharides (LSP1 and LSP2) may be a promising therapy for the treatment of type 2 diabetes mellitus and some related metabolic syndromes.

## Figures and Tables

**Table 1:** Effects of TLSP, LSP1, and LSP2 on FBG.

Treatment	FBG (mmol/L)				
	Day 0	Day 7	Day 14	Day 21	Day 28
Diabetic control	11.88 ± 0.49	13.73 ± 0.56	15.53 ± 0.34	15.39 ± 0.35	15.40 ± 0.65
Rosiglitazone	11.33 ± 0.29	8.82 ± 0.86**	8.08 ± 0.54**	7.48 ± 0.37**	7.23 ± 0.28**
Normal control	5.09 ± 0.12**	5.47 ± 0.96**	5.38 ± 0.82**	5.43 ± 0.85**	5.30 ± 0.55**
TLSP 200 mg/kg	11.57 ± 0.46	8.54 ± 0.26**	7.70 ± 0.45**	7.03 ± 0.41**	6.84 ± 0.37**
TLSP 100 mg/kg	11.56 ± 0.36	8.82 ± 0.61**	7.50 ± 0.50**	7.05 ± 0.46**	6.86 ± 0.68**
LSP1 200 mg/kg	11.69 ± 0.47	8.68 ± 0.45**	7.59 ± 0.27**	7.13 ± 0.53**	7.09 ± 0.25**
LSP1 100 mg/kg	12.1 ± 0.32	9.03 ± 0.52**	7.46 ± 0.30**	7.10 ± 0.43**	7.30 ± 0.59**
LSP2 200 mg/kg	12.17 ± 0.54	8.86 ± 0.48**	7.71 ± 0.33**	7.31 ± 0.23**	7.13 ± 0.40**
LSP2 100 mg/kg	12.67 ± 0.71	9.02 ± 0.60**	7.59 ± 0.24**	7.15 ± 0.30**	7.15 ± 0.29**

Data were presented as mean ± SD (*n* = 7-8). **P < 0.01 versus diabetic control (vehicle-treated).

**Table 2:** Effects of TLSP, LSP1, and LSP2 on OGTT.

Treatment	0 min	30 min	60 min	120 min
Diabetic control	15.68 ± 0.59	25.48 ± 0.66	24.20 ± 0.46	20.30 ± 0.41
Rosiglitazone	8.06 ± 0.27	12.98 ± 0.23**	11.38 ± 0.26**	8.78 ± 0.34**
Normal control	4.72 ± 0.41	9.56 ± 0.27**	8.38 ± 0.34**	5.20 ± 0.43**
TLSP 200 mg/kg	7.70 ± 0.64	12.64 ± 0.43**	10.94 ± 0.36**	8.10 ± 0.50**
TLSP 100 mg/kg	7.48 ± 0.42	12.26 ± 0.43**	11.08 ± 0.30**	8.18 ± 0.41**
LSP1 200 mg/kg	8.70 ± 0.39	14.23 ± 0.33**	12.58 ± 0.34**	9.40 ± 0.28**
LSP1 100 mg/kg	7.42 ± 0.26	12.66 ± 0.33**	11.12 ± 0.22**	8.06 ± 0.11**
LSP2 200 mg/kg	7.62 ± 0.33	12.74 ± 0.38**	11.28 ± 0.50**	8.22 ± 0.35**
LSP2 100 mg/kg	8.78 ± 0.45	14.38 ± 0.66**	13.33 ± 0.68**	9.43 ± 0.33**

Data were presented as mean ± SD (*n* = 7-8). **P < 0.01 versus diabetic control (vehicle-treated).

**Table 3:** Effects of TLSP, LSP1, and LSP2 on FINS and HOMA-IR.

Treatment	FBG (mmol/L)	FINS (mIU/L)	HOMA-IR
Diabetic control	15.40 ± 1.49	46.49 ± 3.15	31.94 ± 2.69
Rosiglitazone	7.12 ± 0.94**	22.93 ± 2.41**	7.26 ± 1.21**
Normal control	5.26 ± 0.41**	9.28 ± 2.11**	2.17 ± 1.11**
TLSP 200 mg/kg	6.84 ± 1.28**	18.81 ± 2.58**	5.72 ± 1.87**
TLSP 100 mg/kg	6.88 ± 1.22**	30.88 ± 1.73**	9.52 ± 1.97**
LSP1 200 mg/kg	7.10 ± 1.28**	20.71 ± 2.73**	6.55 ± 1.37**
LSP1 100 mg/kg	7.42 ± 0.68**	30.51 ± 2.32**	10.06 ± 1.39**
LSP2 200 mg/kg	7.10 ± 1.33**	20.85 ± 2.24**	6.56 ± 2.54**
LSP2 100 mg/kg	7.22 ± 0.96**	31.73 ± 1.96**	10.16 ± 3.01**

Data were presented as mean ± SD (*n* = 7-8). **P < 0.01 versus diabetic control (vehicle-treated).

**Table 4:** Effects of TLSP, LSP1, and LSP2 on serum lipid levels.

Treatment	TC (mmol/L)	TG (mmol/L)	HDL (mmol/L)	LDL (mmol/L)	HDL/TC (%)
Diabetic control	8.09 ± 0.27	2.17 ± 0.45	2.32 ± 0.19	1.63 ± 0.11	28.68 ± 4.03
Rosiglitazone	7.74 ± 0.13	1.55 ± 0.43	2.19 ± 0.51	1.69 ± 0.11	28.29 ± 3.65
Normal control	2.67 ± 0.20	1.26 ± 0.16**	1.70 ± 0.67**	0.15 ± 0.04**	63.67 ± 4.69**
TLSP 200 mg/kg	5.24 ± 0.23**	1.21 ± 0.15**	2.12 ± 0.25**	0.86 ± 0.14**	40.46 ± 3.48**
TLSP 100 mg/kg	6.35 ± 0.48**	1.27 ± 0.16*	2.39 ± 0.23	0.95 ± 0.19**	37.64 ± 3.57**
LSP1 200 mg/kg	5.66 ± 0.22**	1.07 ± 0.12**	2.02 ± 0.3*	0.91 ± 0.12**	35.69 ± 2.82**
LSP1 100 mg/kg	6.41 ± 0.22**	1.24 ± 0.27*	2.53 ± 0.33	1.01 ± 0.08*	39.47 ± 4.11**
LSP2 200 mg/kg	5.73 ± 0.19**	1.26 ± 0.21*	2.52 ± 0.16	1.03 ± 0.06*	43.98 ± 5.72**
LSP2 100 mg/kg	6.26 ± 0.62**	1.03 ± 0.27**	2.5 ± 0.34	0.99 ± 0.08**	39.94 ± 2.62**

Data were presented as mean ± SD (*n* = 7-8). *P < 0.05, **P < 0.01 versus diabetic control (vehicle-treated).

**Table 5:** Effects of TLSP, LSP1, and LSP2 on hepatic glycogen content, G6Pase, and GK activities.

Treatment	Hepatic glycogen content (mg/g)	G6Pase activity (mU)	GK activity (mU)
Diabetic control	16.03 ± 0.12	0.71 ± 0.06	1.06 ± 0.08
Rosiglitazone	10.64 ± 1.13**	0.34 ± 0.07**	3.08 ± 0.46**
Normal control	18.30 ± 0.23**	0.38 ± 0.03**	3.55 ± 0.26**
TLSP 200 mg/kg	17.46 ± 0.36	0.36 ± 0.05**	3.44 ± 0.12**
TLSP 100 mg/kg	18.26 ± 0.04**	0.35 ± 0.02**	3.24 ± 0.21**
LSP1 200 mg/kg	18.11 ± 0.49	0.38 ± 0.02**	3.49 ± 0.14**
LSP1 100 mg/kg	18.92 ± 0.53**	0.38 ± 0.03**	3.36 ± 0.21**
LSP2 200 mg/kg	17.66 ± 1.03	0.37 ± 0.06**	3.36 ± 0.18**
LSP2 100 mg/kg	16.55 ± 0.55	0.39 ± 0.02**	3.31 ± 0.23**

Data were presented as mean ± SD (*n* = 7-8). **P < 0.01 versus diabetic control (vehicle-treated).
